# Support Needs and Coping Strategies as Predictors of Stress Level among Mothers of Children with Autism Spectrum Disorder

**DOI:** 10.1155/2017/8685950

**Published:** 2017-12-25

**Authors:** Sheri R. Kiami, Shelley Goodgold

**Affiliations:** ^1^Department of Physical Therapy, Movement & Rehabilitation Sciences, Northeastern University, 301 Robinson Hall, 360 Huntington Avenue, Boston, MA 02115, USA; ^2^Simmons College, Boston, MA, USA

## Abstract

This study examined maternal stress, coping strategies, and support needs among mothers of children with Autism Spectrum Disorder (ASD). A convenience sample of 70 mothers completed the Parent Stress Index Short Form (PSI-SF), Coping Health Inventory for Parents (CHIP), and Modified Family Needs Questionnaire (FNQ). PSI-SF scores reflected clinically significant levels of stress for 77% of mothers, and mothers identified 62.4% of important needs as unmet. The five most frequently reported important unmet needs were (1) financial support; (2) break from responsibilities; (3) understanding of other after-school program children; (4) rest/sleep; (5) help remaining hopeful about the future. Most coping strategies (81%) were identified as helpful. Additionally, both coping strategies and support needs served as predictors for maternal stress. Maternal stress scores decreased by .402 points for each percent increase in helpful coping strategy, and stress scores increased by .529 points with each percent increase in unmet needs. Given large variation in questionnaire responses across participants and studies, utilization of user-friendly questionnaires, such as the PSI-SF, CHIP, and FNQ, is advocated to determine the evolving important needs unique to each family over the child's lifetime as well as guide prioritization of care, compilation of resources, and referrals for additional services.

## 1. Introduction

With the ever-increasing number of children referred for developmental services, health professionals face larger caseloads and competing demands for direct care, caregiver education, provision of family resources, team meetings, and writing reports. These demands are especially challenging when treating children with Autism Spectrum Disorder (ASD) given its wide constellation of pervasive impairments and their impact on the whole family. ASD is a multidimensional, neurodevelopmental disability affecting social interactions and communication skills [1]. It is characterized by stereotyped repetitive strategies, interests and activities, and common comorbidities that vary in severity across the spectrum including aggression, impulsive behavior, anxiety, depression, sleep difficulties, and cognitive impairments. These impact the child's ability to attain developmental milestones and benefit from educational services.

Additionally, ASD impacts the well-being of the entire family. Parents of children with ASD experience a complex array of stressors that are both directly related to the child's characteristics, for example, difficult behaviors, and indirectly related to system stressors, such as barriers to accessing services for the child [2]. As stressors accumulate, families may experience financial strains, time pressures, marital conflicts, social isolation, increased time caregiving, decreased parenting self-efficacy, insufficient support services, ongoing child-advocacy, and uncertainty about their child's future [3].

Further, sources of caregiver strain change during the child's lifespan, especially during transitions [4]. For example, after the initial diagnosis, caregiver strain may arise from difficulty gathering information on ASD and obtaining early intervention services. During the school years, struggles may shift to accessing special education services and working with a different set of providers each academic year. With the child's transition to adulthood, key stressors may arise from barriers to community access, employment, and housing. Parents may also be faced with challenges related to estate planning, finances, and guardianship. When compared with parents of both typically developing children and children with other disabilities, parents of children with ASD experience greater parenting stress, fatigue, and health problems [5]. Additionally, lower family well-being creates a bidirectional, negative cycle that can exacerbate the child's problem behaviors as well as reducing the positive effects of therapeutic interventions [6–9]. Conversely, improved family functioning can positively impact child outcomes.

Utilization of effective coping strategies has been identified as an important mechanism of managing parental stress and promoting well-being [10, 11]. Hastings et al. [12] described four categories of coping strategies used by parents of children with ASD: (1) active-avoidance (e.g., substance use, self-blame, and venting of emotions); (2) problem-focused (e.g., planning, taking action to deal with problems, and seeking appropriate social supports); (3) positive coping (e.g., using humor, positive reframing, acceptance, and emotional social supports); and (4) religious/denial (e.g., using religion or spirituality, pretending problem does not exist). For mothers of children with ASD, active-avoidance and religious/denial coping strategies have been reported to be maladaptive and linked to decreased well-being and increased depression, anxiety, and stress [11]. In addition, Hastings and Brown [13] found that children's problematic behaviors were associated with the use of maladaptive coping strategies, which is then contributed to increased maternal stress. Furthermore, Hastings et al. [12] found that parents of children with ASD used maladaptive coping methods more often than both parents of typically developing children and parents of children with Down syndrome.

Additionally, assessing and addressing the self-perceived needs of parents of children with ASD not only may be linked to family outcomes but also may enhance the benefits of interventions the child receives [3]. However, there is a paucity of research in the United States on parents' perceptions of important support needs in spite of the potential benefits of family well-being and quality of life [3, 14, 15]. In addition, with the rapidly increasing number of children diagnosed with ASD, addressing family unmet needs may guide prioritization and allocation of limited resources, thereby reducing the burden on healthcare, school, and community services.

A few survey studies on parents' self-reported important needs have been conducted outside the United States. Siklos and Kerns [16] used a modified version of the Waaland et al. Family Needs Questionnaire [17] to compare the needs of parents of children with ASD with parents of children with Down syndrome in British Columbia. The questionnaire lists 54 potential support needs that the participant first self-reports as either “important” or “not important” and then as “met” or “unmet.” Although both groups of parents in their study reported one-third of their important needs as being unmet, specific needs differed. For the parents of children with ASD, the five important needs most frequently rated as unmet were as follows: financial support, my child to have friends, help dealing with fears about my child's future, continuous services, and my other children's friends to feel comfortable around my child. Unmet parent needs were also examined in a more recent Canadian study of 101 parents of children with ASD aged 6–13 years [18]. Two of their most frequently identified unmet needs were similar to those reported by Siklos and Kerns [16], including social inclusion of their child and services provided continuously rather than only in times of crisis.

Examining the support needs of parents residing in a Midwestern US state, Hartley and Schultz [19] reported a higher proportion of important unmet needs for mothers than fathers. Mothers' unmet needs were most often related to the child's social development, family-wide impacts, emotional support, and planning for the future. The authors stated that understanding the important unmet support needs of parents of children with ASD is a critical first step towards improving parent well-being and family outcomes. This, in turn, is widely supported in the literature as a means of promoting better child outcomes [7].

The purpose of this study was, therefore, to better understand support needs and coping strategies among mothers of children with ASD, and how these factors may predict maternal stress. Specifically, we hypothesized that mothers would report high levels of stress as well as high levels of important unmet support needs. Further, we hypothesized that there would be an increase in maternal stress as important unmet needs increased, and a decrease in maternal stress as the utilization of helpful coping strategies increased. With greater understanding of the relationships among maternal stress, unmet needs, and coping strategies, healthcare professionals can better prioritize care within their scope of practice, compile key resources, and provide referrals for additional support services.

## 2. Methods

### 2.1. Participants

A convenience sample of mothers with children diagnosed with autism was recruited in-person during local parent meetings, educational events, and conferences hosted by four Boston-area organizations known to serve families of children with ASD, including the Asperger's Association of New England, Federation for Children with Special Needs, Boston Families for Autism/Towards Independent Living, and the Special Education Parent Advisory Council of the Boston Public Schools. Our rational reason for including only mothers was trifold: (1) mothers more frequently have the primary role of caregiver for children; (2) mothers experience higher level of stress; and (3) mothers have not only higher levels of unmet needs but types of needs which differ between mothers and fathers [2, 3, 15, 19, 20].

Data were collected via anonymous survey packets with stamped, self-addressed return envelopes. Each packet contained a cover letter explaining the purposes of the study, introducing the researchers, notifying mothers of their rights and that participation in the study was voluntary. All participants read and signed an informed consent to participate in this research. To preserve anonymity, participants were instructed to exclude their names from the questionnaires, and completed questionnaires were immediately separated from the signed consent and raffle forms upon receipt.

Since recruitment of participants was in-person and anonymous, neither reminder letters nor a second survey packet could be mailed to enhance the return rate. Therefore, to incentivize participants, mothers could choose to enter a raffle for one of five $25.00 gift certificates. To maintain survey anonymity, raffle forms were immediately removed and kept separate from the returned survey packets. Study inclusion criteria were self-identification as a mother of a child with a diagnosis of ASD and the ability to read English.

Consistent with survey studies of this population published in the literature [16, 18, 21], the return rate of completed packets was 24% (70 packets out of 290 packets distributed).

### 2.2. Measures

 Each packet included a demographic questionnaire developed by the researchers to identify participant characteristics (Table 1). To provide a measure of the child's everyday executive function, mothers also completed the Behavior Rating Inventory of Executive Function (BRIEF). *T* scores of 65 or above on the BRIEF indicate clinically significant behavioral dysfunction. For families with two or more children with ASD, mothers were instructed to choose one child and answer all questions with reference to that child only.

Packets also included the Parent Stress Index Short Form (PSI-SF) developed by Abidin [22] to measure maternal stress level, the Modified Family Needs Questionnaire (FNQ) developed by Siklos and Kerns [16] to identify parent self-reported important unmet needs, and the Coping Health Inventory for Parents (CHIP) by McCubbin et al. [23] to identify parent coping strategies and a self-addressed, stamped envelope.

#### 2.2.1. Maternal Level of Stress

The PSI-SF, a derivative of the original tool used to measure parental stress levels [22], was developed to reduce the nearly prohibitive time required for typical assessment batteries. The short form is a 36-item questionnaire comprised of three subscales: parental distress, parent-child dysfunctional interaction, and difficult child. The parental distress (PD) subscale yields a score that indicates level of distress resulting from personal factors, such as depression or conflict with a partner and from life restrictions due to the demands of childrearing. The parent-child dysfunctional interaction (P-CDI) subscale provides an indication of the parent's dissatisfaction with interactions with their child and the degree to which the parent finds their child's strategies unacceptable. The difficult child (DC) subscale measures the parent's perceptions of their child's self-regulatory abilities. Each subscale consists of 12 items, and the entire short form takes about 10 minutes to complete. Validation of the short form was based on two samples of mostly white married mothers of young children, and the correlation coefficient on the short form was acceptably high at 87%.

#### 2.2.2. Maternal Coping Strategies

The* Coping Health Inventory for Parents (CHIP) *is a 45-item questionnaire designed to identify coping strategies utilized by parents of children with serious or chronic health conditions [23]. Parents are instructed to circle the coping strategies they use and to rate its helpfulness on a 4-point scale: (3) extremely helpful, (2) moderately helpful, (1) minimally helpful, and (0) not helpful. In addition, for each coping behavior not used, the parent is asked to identify “chose not to use it” or “not possible” to use. The CHIP total score is the sum of points for all 45 items. The researchers reported that each of the subscales has shown adequate internal reliability (Cronbach's alphas = 0.79, 0.79, and 0.71, resp.) and validity. Rasch analysis, however, supported only two of the 3 subscales [24], and collapsing response categories was recommended.

#### 2.2.3. Family Needs

The modified FNQ is a 54-item questionnaire modified by Siklos and Kerns [16] to examine the needs among parents of children with ASD and Down syndrome. It includes 23 “I need” statements from the original FNQ [17] and 31 new statements. In addition, in instances when the support benefit was clearly directed solely for the parent or for the child, need statements were also identified as parent-centered or child-centered. Cronbach's alpha coefficients ranging from 0.78 to 0.89 were reported as providing evidence of acceptable reliability. To investigate healthy lifestyle strategies, we added three self-care need statements related to the mother's health, participation in physical activity, and nutrition.

The FNQ utilizes a 4-point scale to indicate the degree that the need is important to the respondent: (1) not important, (2) slightly important, (3) important, or (4) very important. In addition, for each need statement, the respondent chooses “Yes,” “Partly,” or “No” to indicate if the need is currently being met. Responses are then classified as “Important” if respondents identify an “I need” statement as either “Important” or “Very Important” and “Unmet” if identified as either “Partly Met” or “Unmet.”

### 2.3. Procedure and Data Analysis

The Institutional Research Board at the authors' academic institution reviewed and approved this study prior to participant recruitment. As survey packets were returned, the researchers scored individual surveys in accordance with each tool's administration manual, and scores were entered into Excel spreadsheets. To validate scoring and prevent data entry errors, a second researcher scored and checked data entry for 10% of participants via random sampling. Data were then exported to an IBM SPSS Statistics 23 software program for analyses.

#### 2.3.1. Descriptive Statistics and Scoring

Descriptive statistics were used to analyze participants' demographics and questionnaire responses.

The PSI-SF utilizes a 5-point scale to indicate the degree to which the parent agrees with each of the 36 statements, from 5 (strongly agree) to 1 (strongly disagree.) Each subscale raw score was calculated by summing the points of the 12 items in that subscale. Higher raw scores reflect greater stress. The sum of the three subscales yields a total raw score, which was then converted into a percentile score based on a chart provided on the PSI-SF scoring sheet. PSI-SF percentile scores of 85% or above are identified as high, and scores of 90% or above reflect clinically significant levels of stress.

CHIP coping data for all analyses in this study were based on the sum of the three subscales and collapsed response categories of either helpful (extremely or moderately helpful) or not helpful (minimally or not helpful). Percentages of helpful coping strategies were then calculated by dividing the total score by the number of coping strategies identified as helpful (helpful coping strategy percentage = number of helpful coping strategies/total of coping strategies utilized).

FNQ support needs data used in our study were calculated in two ways. For need statements, we calculated the sums of participants identifying each statement as important or not important and as met or unmet. Percentages of important needs were calculated by dividing the sum of needs identified as important by the total number of need statements (important needs percentage = number of need statements identified as important/total number of need statements). In addition, need statements identified as both important and unmet were summed, and percentages were calculated by dividing the sum of important unmet needs by the sum of all important needs (important unmet needs percentage = number of important needs identified as unmet/total number of important needs). Similar calculations were also performed for individual participants.

#### 2.3.2. Linear Regression

IBM SPSS Statistics 23 software program for linear regression was used to predict participants' stress levels based on percentages of helpful coping strategies and on percentages of important unmet needs and to predict the percentages of unmet support needs based on percentages of helpful coping strategies. In addition, after centering the data and creating an interaction term, SPSS linear regression was used to perform a moderator analysis to examine if the percent of helpful coping strategies altered the strength of the relationship between important unmet needs and maternal stress. Level of significance was set at 0.05 for all analyses.

## 3. Results

### 3.1. Descriptive Results

#### 3.1.1. Demographics

A total of 70 mothers raising a child with ASD participated in this study. Two-thirds of the participants were white, non-Hispanic, and the majority of respondents identified an income bracket of $50,000 or higher per year (Table 1). These demographic characteristics are similar to those reported for the general population of children with a diagnosis of ASD [25].

#### 3.1.2. Maternal Stress

PSI-SF mean raw scores for total stress (106.15; SD 22.57) and for mean raw scores of subscales P-CDI (29.52; SD 8.99) and DC (41.25; SD 9.72) when converted into percentile scores reached levels indicative of clinically significant levels of stress. The PD subscale raw mean (35.38, SD 8.74) when converted into a percentile score was considered high but missed the clinically significant level of stress by less than one point.

The total PSI-SF percentile scores reflected a clinically significant level of stress for 77% of the mothers participating in this study. For two of the subscales, clinically significant levels of stress were also found for the majority of participants, 63% of participants on the P-CDI Subscale, and 68% of participants on the DC Subscale. For the PD subscale, 48% of scores reflected a clinically significant level of distress.

#### 3.1.3. Coping Strategies

A mean of 33 coping strategies were utilized by mothers, and 81% of these were reported to be helpful. The five most frequently reported coping strategies are presented in Table 2. “Believing that my child will get better” was reported as the most helpful coping strategy that our participants utilized. “Explaining our family situation to friends and neighbors so they will understand” and “eating” were the two coping strategies reported as least helpful. The least frequently utilized coping strategy was “Going out with my spouse on a regular basis.”

#### 3.1.4. Family Needs

FNQ data were analyzed for each mother and for each need statement.  Table 3 presents percentages of self-reported “important needs” and “important unmet needs” for each need statement. FNQ statement order is ranked by percentage from most to least frequently reported need identified as both important and unmet (far right column). Participants reported an average of 47.3 of the 57 (83%) need statements as “important.” Percentages of important needs ranged from a high of 100% to a low of 49%, and all of the need statements except for two were rated as “important” by at least 65% of the participants.

On average, 62.4% of the need statements identified as important were also reported as unmet. Percentages of important unmet needs ranged from a high of 87% to a low of 25%, with a standard deviation of 23.7%. Important unmet needs that were reported by over 75% of mothers included (1) financial support for the child's therapies, treatment, and care; (2) a break from responsibilities; (3) having other after-school program children understand the child's special needs; (4) getting enough rest or sleep; (5) help remaining hopeful about the child's future; (6) counseling for the mother and her significant other; (7) family to understand the child's problems; and (8) time to care for the mother's own health needs. None of these items are categorized as child-centered.

Percentages of important needs that were identified as met ranged from a high of 75% to a low of 12%. The only important need reported as met by 75% of mothers was “to have professionals working with my child speak to me in terms I can understand.”

### 3.2. Linear Regression Results

#### 3.2.1. Helpful Coping Strategies as a Predictor of Maternal Stress

Linear regression using the PSI-SF total raw score as the dependent variable and the percent of helpful coping strategies as the predictor variable revealed an inverse relationship, with a *β* coefficient of −0.403 (se = .122; *t* = −3.317; *p* = .001). That is, maternal stress score decreased .403 points for each percent increase in helpful coping strategies.

#### 3.2.2. Important Unmet Needs as a Predictor of Maternal Stress

Linear regression analysis using the PSI-SF total raw score as the dependent variable and the percent of important unmet needs as the predictor variable revealed a *β* coefficient of .529 (se = .099; *t* = 5.138; *p* = .000). That is, maternal stress score increased .529 percentage points for each percent increase in important unmet needs.

#### 3.2.3. Helpful Coping Strategies as a Predictor of Important Unmet Needs

Linear regression analysis using the percent of important unmet needs as the dependent variable and the percent of helpful coping strategies as the predictor variable revealed and inverse relationship, with a *β* coefficient of −.52 (se = .176; *t* = −2.950; *p* = .004). That is, important unmet needs decreased .52 percentage points for each percent increase in helpful coping strategies. However, when a linear regression analysis was performed to examine helpful coping strategies as a moderator for the strength of the relationship between important unmet needs and maternal stress, the interaction failed to reach statistical significance.

## 4. Discussion

The purpose of this study was to better understand support needs and coping strategies among mothers of children with ASD and how these factors may predict maternal stress level. A convenience sample of 70 mothers completed the PSI-SF to identify maternal stress level, the modified FNQ to identify important unmet support needs, and the CHIP to identify helpful coping strategies.

### 4.1. Maternal Stress

Consistent with previous studies [3, 15], the majority of mothers (77%) in our study presented with clinically significant levels of stress. Interventions for reducing high levels of maternal stress have often focused on training parents to better regulate the child's challenging strategies [3, 13, 15]. This approach, however, is narrow in scope and fails to address the social-emotional impact of parenting a child with ASD. In contrast, more recent models of stress for parents of children with ASD include family resources and support needs as intervening variables that may buffer or improve both family well-being and child outcomes [2, 3, 7, 15].

### 4.2. Maternal Important Unmet Needs

 More than half of the needs identified as important by mothers in our study, 62.4%, were also reported as unmet. Hartley and Schultz [19] advocate that understanding parents' important unmet needs is a critical first step in prioritizing system-wide services. Our results highlight the prevalence of unmet needs among mothers of children with ASD and are consistent with previous research findings by Siklos and Kerns [16] and Brown et al. [18]. It is unclear whether our findings reflect the lack of available resources in the community or reflects knowledge, access, or utilization of support services. Nonetheless, these results reveal that, for many families, the service delivery system is failing to adequately address their needs.

Notably, the range of important unmet needs reported by mothers in this study varied widely, as evidenced by a large standard deviation of nearly 24%. Comparisons with other research studies are difficult since standard deviations were often not reported [16, 18, 19]. In addition, there is marked variation in the percentages of participants reporting each FNQ statement. For example, there are substantially lower percentages of maternal important unmet needs reported in the Hartley and Schultz study [19]. Their highest reported important unmet need, “my child to have friends of his/her own,” was only 37% compared with our highest important unmet need, “financial support,” at 87%. Financial support was not even among their five most frequently reported important unmet needs. Although financial support was the most frequently reported important unmet need in our study and the study by Siklos and Kerns [16], our other nine most frequently reported important unmet needs differed.

Variations in results among studies are not easily explained. Beyond the uniqueness of each child with ASD, family composition, and demographic characteristics, all of the studies employed convenience samples with inherent response bias. Further, differences across studies cannot be explained by the inclusion of fathers as participants. Hartley and Schultz's [19] data on fathers and mothers are presented separately, and over 90% of participants in the other two studies were mothers [16, 18]. Also, generalizability of the results to other jurisdictions is cautioned, since funding and school services were not reported. These variations across study participants underscore the importance of individual assessment of each unique family's important unmet needs. In addition, ongoing reassessment is advocated particularly during transitions as needs change, and the child and family face different demands and expectations.

### 4.3. Helpful Coping Strategies as a Predictor for Maternal Stress

As hypothesized, percent of helpful coping strategies was found to predict maternal stress level. Increases in helpful coping behavior were associated with decreases in maternal stress level. Similar to other study findings [26], participants in our sample utilized a wide variety of adaptive coping strategies that were problem-focused, for example, investing myself in my child, and that reflected positive thinking, for example, believing my child will get better.

The three coping strategies most frequently reported as not helpful were as follows: explaining the family situation to others, eating, and getting angry. None of these strategies tackle the challenges of raising a child with ASD directly and may therefore be categorized as maladaptive. Professionals are, therefore, encouraged to examine the strategies mothers are utilizing to help cope with high parenting demands and stress and to foster use of adaptive strategies.

### 4.4. Unmet Needs as a Predictor for Maternal Stress

As hypothesized, the percentage of important unmet needs was found to predict maternal stress level. Increases in important unmet needs were associated with increases in maternal stress level. This finding supports recommendations that healthcare practitioners seek opportunities to address family unmet needs. For mothers of children with ASD, however, other factors have also been associated with maternal stress level. For example, parental self-efficacy, belief in one's own ability to effectively assume the role of parenting, has been linked to the child's behavior problems and maternal anxiety and depression [3, 13, 15, 27].

### 4.5. Practice Implications

Pediatric healthcare practitioners widely endorse family-centered care (FCC) as a best practice [4, 15, 28]. This approach may be particularly critical when working with families raising children with ASD given the pervasive nature of the disability and bidirectional relationship between child outcomes and family well-being [3, 7, 15]. Although parental self-efficacy, coping strategies, and other parenting characteristics are important components for family well-being, the substantial demands associated with raising a child with ASD necessitate continued support from external sources [3]. Within the family-centered delivery model, pediatric services address both child and family needs and preferences, and professionals build family competency and capacity through interventions, resources, and supports [7]. One of the greatest challenges for pediatric service providers, however, is concurrently addressing the unique evolving needs of both the child and family.

Given the high prevalence and wide variation in identification of important unmet needs across participants in our study as well as across research studies, individual assessment of family support needs is advocated. Additionally, assessments should be ongoing as demands, responsibilities, and needs shift with transitions during the child's lifetime. Russa and colleagues [4] caution that even when professionals address needs in one developmental phase, caregivers' needs may shift from learning about the child's diagnosis to the child's special education rights, to resources for health insurance, recreation or respite, and then, with the child's transition to adulthood, to access for adult services.

Pediatric practitioners are well positioned to routinely screen mothers for high levels of stress, coping strategies, and important support needs using the PSI-SF, CHIP, and FNQ or other valid measures. These tools are low in cost, require little time to complete, and are self-administered. This avoids using therapist time that otherwise would have been available for direct child care and allows caregivers to complete the questionnaires at their convenience. Further, parents are often so overwhelmed or worried about their child's future that they cannot pinpoint specific desired child or family goals when asked verbally. In these instances, questionnaire items may help parents recognize problems their child and family are encountering, which in turn can facilitate their ability to identify specific child intervention goals that can enhance family functioning and well-being. As a result, positive child outcomes are believed to be more likely [3, 14, 18, 19].

Caregiver questionnaire responses can be used to prioritize and guide direct interventions that are within the clinician's scope of practice as well as compile resources. For example, depending on the parent's identified important unmet needs, pediatric practitioners can train parents to promote specific developmental skills or manage the child's difficult behaviors, help create family routines, plan outings to reduce social isolation, promote helpful coping strategies and parental self-efficacy, advise participation in healthy lifestyle strategies and other self-care activities that reduce stress and promote well-being, support teamwork, obtain access to therapies, and provide resources for support services, such as parent support groups, community recreation activities, or respite care. Practitioners are encouraged to refer to other healthcare professionals when parental stress and unmet needs are identified that are outside their scope of practice.

### 4.6. Study Strengths, Limitations, and Suggestions for Future Studies

One methodological strength in this study was the use of a support needs questionnaire that elucidates both whether the need is important to the respondent and whether each important need is met or unmet. Another strength is that the CHIP and FNQ-SF can be used readily by practitioners, are low in cost, require little time to complete, and are easy for family members to self-administer.

This study, however, has several methodological limitations. First, the small sample size and participation rate (24%) limit generalizability of our findings to parents of children with ASD in other geographical locations and with different demographics. Second, given the small sample size, we were unable to statistically account for differences related to the child's age or symptom severity. In addition, participants were mothers only, whose perceptions of unmet needs vary somewhat from those of fathers [19]. Further, this study examined only two variables related to maternal stress, and other factors, such as the child's problem behaviors, area resources, or parenting characteristics, may also produce reciprocal effects on child and family outcomes.

Future research is recommended to examine the underlying causes that produce such high percentages of important unmet needs and to examine the effectiveness of pediatric interventions on both child and caregiver outcomes. Studies on the feasibility of implementing family assessments that measure well-being are also needed. Even in organizations that endorse FCC, there are many barriers. Time is often reported as the greatest barriers, but other common barriers include a lack of training in FCC, lack of knowledge of resources within the community that might meet the family's needs, resistance to shifting to shared decision-making, meeting productivity levels, and lack of funding or insurance for services outside of direct care within the professional's scope of practice [14, 29–31].

## 5. Conclusions

Our findings further elucidate the relationships among maternal stress level, coping strategies, and support needs. Increases in helpful coping strategies utilized by mothers were associated with lower levels of maternal stress, but increases in important unmet needs were associated with increased levels of maternal stress. Given the high incidence of reported unmet needs and large variation across participants and studies, utilization of user-friendly questionnaires, such as the PSI-SF, CHIP, and FNQ, is advocated to determine the evolving important needs unique to each family over the child's lifetime. This may enhance the ability of pediatric healthcare practitioners to guide prioritization of direct care within the clinician's scope of practice, compile resources, and refer to other healthcare professionals when parent stress and unmet needs are identified that are outside their scope of practice.

## Figures and Tables

**Table 1:** Demographic characteristics of the sample (*N* = 70).

Characteristics	*n* (%)
Race/ethnicity of mother	
Hispanic	4 (6)
White not Hispanic	55 (77)
Black not Hispanic	8 (11)
Others	3 (5)
Primary caregiver in home	
Mother	66 (94)
Other family/household members	3 (4)
Hired childcare provider	1 (1)
Income bracket	
Less than $15,000	6 (9)
$15,000– $24,999	3 (4)
$25,000–$49,999	10 (14)
$50,000–$74,999	10 (14)
$75,000–$99,999	15 (22)
$100,000 & over	26 (38)
Number of children in home: mean (SD)	2.1 (1.12)
Number of children in home with special needs: mean (SD)	1.34 (0.63)
Age of child with ASD (years): mean (SD)	9.7 (5.5)
Child with ASD BRIEF^*∗*^ score: mean (SD)	70 (12)
Child with ASD school placement	
Public school placement	57 (81)
Private School placement	8 (11)
Does not attend school or daycare	3 (4)
Residential placement	2 (3)

^*∗*^BRIEF: Behavior Rating Inventory of Executive Function *T* score of 65 or above indicates clinically significant behavioral dysfunction.

**Table 2:** Most and least frequently reported coping strategies.

Coping strategy	*n*	%
Most frequently reported as extremely helpful.		
(1) Believing that my child will get better.	41	59.4
(2) Investing myself in my child.	37	53.6
(3) Having my child with special needs seen by professionals on a regular basis.	36	52.2
(4) Talking with other parents in the same situation and learning about their experiences.	33	47.8
(5) Doing things together as a family, including all members of the household.	32	46.4
Most frequently reported as* not *helpful.		
(1) Explaining our family situation to friends and neighbors so they will understand; eating. (Tied)	23	33.3
(2) Allowing myself to get angry.	21	30.4
(3) Encouraging my child with special needs to be more independent.	19	27.5
(4) Getting other members of the family to help with chores and tasks at home.	17	24.6
(5) Believing that things will always work out; telling myself that I should be thankful for. (Tied)	13	18.8
Least frequently reported as utilized		
(1) Going out with my spouse on a regular basis.	32	46.4
(2) Entertaining friends in my home.	31	44.9
(3) Concentrating on hobbies (art, jogging, etc.).	29	42.0
(4) Doing things with family relative; getting away by myself; and working outside employment; investing time & energy in my job (3-way tie).	25	36.2
(5) Purchasing gifts from myself/others.	24	34.8

**Table 3:** Family needs questionnaire percentages ranked by important unmet needs.

“I need” statement^a^	% important unmet needs	% important needs
Financial support in order to provide my child with therapies, treatments, and care. (n/a)	87%	89%
To get a break from my responsibilities. (P)	86%	94%
To have the other children in my child's after-school program understand my child's special needs. (n/a)	79%	100%
To get enough rest or sleep. (P)	79%	96%
Help remaining hopeful about my child's future. (P)	79%	85%
To have counseling for myself and my spouse/partner/child's father. (P)	78%	69%
To have other family members understand my child's problems. (n/a)	77%	94%
To have time to care for my own health needs (Note: additional question added to Modified FNQ. (P)	76%	90%
Weekend and after-school activities for my child with special needs. (C)	74%	87%
To be told if I am making good decisions about my child. (P)	73%	82%
To be physical active or exercise. (P)	73%	96%
To be shown what to do when my child is acting unusually or is displaying difficult strategies. (P)	72%	93%
To have my child's therapies continue throughout the summer months and school breaks. (C)	72%	97%
Help dealing with my fears about my child's future. (P)	71%	81%
To have help in deciding how much to let my child do by himself/herself. (P)	71%	80%
To spend time with my friends. (P)	71%	84%
To have help from family members taking care of my child. (P)	70%	78%
Information about special programs and services available to my child and my family. (n/a)	70%	96%
For my child to have friends of his/her own. (C)	70%	94%
To have time to spend alone with a spouse/partner/companion. (P)	70%	90%
For my child with special needs to have social activities other than with his/her own parents and siblings. (C)	67%	93%
To have consistent behavioral therapy for my child. (C)	65%	96%
To have help with housework. (P)	65%	76%
To be encouraged to ask for help. (P)	65%	82%
To be reassured that it isn't uncommon to have negative feelings about my child's strategies. (P)	64%	65%
To be told why my child acts in ways that are different, difficult or unusual. (P)	64%	79%
Q To have my questions answered honestly. (P)	64%	97%
For my other children's friends to feel comfortable around my child. (n/a)	63%	88%
To have different professionals agrees on the best way to help my child. (n/a)	61%	93%
The children in my child's classroom to understand that my child cannot help his/her unusual strategies and difficulties. (n/a)	61%	80%
For professionals working with my child to understand my child's and family's needs. (n/a)	60%	94%
To have my child's teacher(s) understand his/her special needs. (P)	58%	93%
To have time to spend alone with my other children. (n/a)	57%	100%
My child's doctor and dentist to have experience working with children like my child. (n/a)	56%	97%
To eat nutritious meals. (P)	55%	96%
To have counseling for my other children. (n/a)	55%	73%
To take a family vacation each year. (n/a)	54%	88%
To work with professional with experience with child similar to my child. (C)	54%	93%
To have professional to turn to for advice or services when my child needs help. (P)	54%	96%
To have consistent speech therapy for my child. (C)	53%	85%
To take a vacation without my child with special needs each year. (n/a)	49%	65%
To be shown that my opinions are used in planning my child's treatment, therapies or education. (P)	49%	97%
To have information regarding my child's therapeutic or educational progress. (P)	47%	100%
Q. Respite care for my child. (P)	46%	58%
Continuous services rather than only in times of crisis. (C)	46%	93%
To be actively involved in my child's treatment and therapies. (P)	44%	99%
For professionals to be discrete when talking about my child while he/she is in the room. (C)	43%	71%
My child to have a teacher's aide with him/her at school who has knowledge or experience working with children like my child. (C)	42%	83%
To go out to dinner with my family. (n/a)	41%	77%
To have my child's teacher(s) understand his/her special needs. (C)	40%	94%
To be well educated about my child's disorder so I can be an effective decision maker regarding the needs of my child. (P)	39%	100%
To have consistent occupational therapy for my child. (C)	38%	81%
To have my spouse/partner/child's father agree on decisions about our child with special needs. (P)	36%	89%
My child's school to set up a specialized education plan for my child. (C)	32%	100%
To have consistent physical therapy for my child. (C)	30%	49%
To be shown respect by the professional working with my child. (P)	28%	97%
To have professional working with my child to speak to me in terms I can understand. (P)	25%	97%

“I need” statement^a^ (P) indicates parent-focused need; (C) indicates child-focused need; (n/a) indicates neither parent- nor child-focused need.

## References

[1] American Psychiatric Association (2013). *Diagnostic and Statistical Manual of Mental Disorders*.

[2] Bluth K., Roberson P. N., Billen R. M., Sams J. M. (2013). A stress model for couples parenting children with autism spectrum disorders and the introduction of a mindfulness intervention. *Journal of Family Theory & Review*.

[3] Karst J. S., van Hecke A. V. (2012). Parent and family impact of autism spectrum disorders: a review and proposed model for intervention evaluation. *Clinical Child and Family Psychology Review*.

[4] Russa M. B., Matthews A. L., Owen-DeSchryver J. S. (2015). Expanding Supports to Improve the Lives of Families of Children With Autism Spectrum Disorder. *Journal of Positive Behavior Interventions*.

[5] Hayes S. A., Watson S. L. (2013). The impact of parenting stress: A meta-analysis of studies comparing the experience of parenting stress in parents of children with and without autism spectrum disorder. *Journal of Autism and Developmental Disorders*.

[6] Baker J. K., Smith L. E., Greenberg J. S., Seltzer M. M., Taylor J. L. (2011). Change in Maternal Criticism and Behavior Problems in Adolescents and Adults With Autism Across a 7-Year Period. *Journal of Abnormal Psychology*.

[7] Dunst C. J., Trivette C. M., Hamby D. W. (2007). Meta-analysis of family-centered helpgiving practices research. *Mental Retardation and Developmental Disabilities Research Reviews*.

[8] Mandell D. S., Xie M., Morales K. H., Lawer L., McCarthy M., Marcus S. C. (2012). The interplay of outpatient services and psychiatric hospitalization among medicaid-enrolled children with autism spectrum disorders. *Archives of Pediatric and Adolescent Medicine*.

[9] Osborne L. A., McHugh L., Saunders J., Reed P. (2008). Parenting stress reduces the effectiveness of early teaching interventions for autistic spectrum disorders. *Journal of Autism and Developmental Disorders*.

[10] Ludlow A., Skelly C., Rohleder P. (2012). Challenges faced by parents of children diagnosed with autism spectrum disorder. *Journal of Health Psychology*.

[11] Seymour M., Wood C., Giallo R., Jellett R. (2013). Fatigue, stress and coping in mothers of children with an autism spectrum disorder. *Journal of Autism and Developmental Disorders*.

[12] Hastings R. P., Kovshoff H., Brown T., Ward N. J., Espinosa F. D., Remington B. (2005). Coping strategies in mothers and fathers of preschool and school-age children with autism. *Autism*.

[13] Hastinqs R. P., Brown T. (2002). Behavior problems of children with autism, parental self-efficacy, and mental health. *American Journal on Intellectual and Developmental Disabilities*.

[14] Hodgetts S., Zwaigenbaum L., Nicholas D. (2014). Profile and predictors of service needs for families of children with autism spectrum disorders. *Autism*.

[15] Kuhaneck H. M., Madonna S., Novak A., Pearson E. (2015). Effectiveness of interventions for children with autism spectrum disorder and their parents: A systematic review of family outcomes. *American Journal of Occupational Therapy*.

[16] Siklos S., Kerns K. A. (2006). Assessing need for social support in parents of children with autism and Down syndrome. *Journal of Autism and Developmental Disorders*.

[17] Waaland P. K., Burns C., Cockrell J. (1993). Evaluation of needs of high- and low-income families following paediatric traumatic brain injury. *Brain Injury*.

[18] Brown H. K., Ouellette-Kuntz H., Hunter D., Kelley E., Cobigo V. (2012). Unmet Needs of Families of School-Aged Children with an Autism Spectrum Disorder. *Journal of Applied Research in Intellectual Disabilities*.

[19] Hartley S. L., Schultz H. M. (2014). Support Needs of Fathers and Mothers of Children and Adolescents with Autism Spectrum Disorder. *Journal of Autism and Developmental Disorders*.

[20] Abbeduto L., Seltzer M. M., Shattuck P., Krauss M. W., Orsmond G., Murphy M. M. (2004). Psychological Well-Being and Coping in Mothers of Youths with Autism, Down Syndrome, or Fragile X Syndrome. *American Journal on Intellectual and Developmental Disabilities*.

[21] Hodgetts S., Nicholas D., Zwaigenbaum L., McConnell D. (2013). Parents' and professionals' perceptions of family-centered care for children with autism spectrum disorder across service sectors. *Social Science & Medicine*.

[22] Abidin R. R. (1995). *Manual for the Parenting Stress Index*.

[23] McCubbin H. I., McCubbin M. A., Patterson J. M., Cauble A. E., Wilson L. R., Warwick W. (1983). CHIP-Coping Health Inventory for parents: an assessment of parental coping patterns in the care of the chronically ill child. *Journal of Marriage and Family*.

[24] Gothwal V. K., Bharani S., Reddy S. P. (2015). Measuring coping in parents of children with disabilities: A Rasch model approach. *PLoS ONE*.

[25] Wingate M., Kirby R. S., Pettygrove S. (2014). Prevalence of autism spectrum disorder among children aged 8 years-autism and developmental disabilities monitoring network, 11 sites, United States, 2010. *Morbidity and Mortality Weekly Report Surveillance Summaries*.

[26] Lai W. W., Oei T. P. S. (2014). Coping in Parents and Caregivers of Children with Autism Spectrum Disorders (ASD): a Review. *Review Journal of Autism and Developmental Disorders*.

[27] Rezendes D. L., Scarpa A. (2011). Associations between parental anxiety/depression and child behavior problems related to autism spectrum disorders: the roles of parenting stress and parenting self-efficacy. *Autism Research and Treatment*.

[28] Kuo D. Z., Houtrow A. J., Arango P., Kuhlthau K. A., Simmons J. M., Neff J. M. (2012). Family-centered care: current applications and future directions in pediatric health care. *Maternal and Child Health Journal*.

[29] O'Neil M. E., Palisano R. J., Westcott S. L. (2001). Relationship of therapists' attitudes, children's motor ability, and parenting stress to mothers' perceptions of therapists' behaviors during early intervention. *Physical Therapy in Sport*.

[30] Christon L. M., Myers B. J. (2015). Family-centered care practices in a multidisciplinary sample of pediatric professionals providing autism spectrum disorder services in the United States. *Research in Autism Spectrum Disorders*.

[31] Fingerhut P. E., Piro J., Sutton A. (2013). Family-centered principles implemented in home-based, clinic-based, and school-based pediatric settings. *American Journal of Occupational Therapy*.

